# Serial and parallel organ‐at‐risk‐specific noncoplanar arc optimization for small versus large target volumes in liver SBRT

**DOI:** 10.1002/acm2.14396

**Published:** 2024-06-18

**Authors:** John Lincoln, Lee MacDonald, Lucy Ward, Shelly Johnston, Alasdair Syme, Christopher Thomas

**Affiliations:** ^1^ Department of Physics and Atmospheric Science Dalhousie University Halifax Canada; ^2^ Department of Radiation Oncology Dalhousie University Halifax Canada; ^3^ Department of Medical Physics Nova Scotia Health Halifax Canada; ^4^ Department of Radiology Dalhousie University Halifax Canada

**Keywords:** liver SBRT, noncoplanar, parallel OARs

## Abstract

Noncoplanar arc optimization has been shown to reduce OAR doses in SRS/SRT and has the potential to reduce doses to OARs in SBRT. Extracranial targets have additional considerations, including large OARs and, in the case of the liver, volume constraints on the healthy liver. Considering pathlengths through OARs that encompass target volumes may lead to specific dose reductions as in the encompassing healthy liver tissue. These optimizations must also leverage delivery efficiency and trajectory sampling to ensure ease of clinical translation. The purpose of this research is to generate optimized static‐couch arcs that separately consider serial and parallel OARs and arc delivery efficiency, with a trajectory sampling metric, towards the aim of reducing dose to OARs and the surrounding healthy liver tissue. Separate BEV cost maps were created for parallel, and serial OARs by means of a fast ray‐triangle intersection algorithm. An additional BEV cost map was created for the liver which, by definition, encompasses the liver tumors. The individual costs of these maps were summed and combined with the sampling metric for 100 000 random combinations of arc trajectories. A search algorithm was applied to find an arc trajectory solution that satisfied BEV cost and sampling optimization, while also ensuring an efficient delivery was possible with a low number of arcs. This method of arc selection was evaluated for 16 liver SBRT patients characterized by small and large target volumes. Comparisons were made with a clinical arc template of coplanar arcs. Dosimetric plan quality was evaluated using published guidelines and metrics from RTOG1112. Four of five plan quality metrics for the liver were significantly reduced when planned with optimized noncoplanar arcs. Median (range) reductions of the volumes receiving 10, 18, and 21 Gy were found of 140.4 (295.8) cc (*p* = 0.001), 28.2 (230.6) cc (*p* = 0.002) and 18.5 (155.5) cc (*p* = 0.04). A significant increase in median (range) dose to the right kidney of 0.2 ± 0.9 Gy (*p* = 0.03) was also found using optimized noncoplanar arcs, which was below the tolerance of 10 Gy for all cases. The average number of arcs chosen was 4 ± 1. Optimizing serial and parallel OARs separately during static couch noncoplanar arc selection significantly reduced the dose to the liver during SBRT using a moderate number of arcs.

## 1 INTRODUCTION

Noncoplanar optimization methodologies are a subset of trajectory optimization techniques that aim to leverage additional degrees of freedom in radiotherapy (RT) treatment settings.^1^ C‐arm linear accelerators have great potential for trajectory optimization due to the many axes available for manipulation. Advances in the last decade of research have progressed from beam angle optimization (BAO) that automatically selects ports for intensity‐modulated radiotherapy (IMRT),^2^, ^3^, ^4^, ^5^ to efficiently choreographed nonisocentric dynamic couch translation and rotation techniques, where almost all possible axes are optimized.^6^ Throughout this time, retrospective comparisons between these increasingly complex techniques and the clinically accepted reliability of volumetric modulated arc therapy (VMAT),^7^ have not been significant enough to realize widespread clinical adoption of these technologies.

In one prospective clinical trial, noncoplanar IMRT beams were used in a cohort of 11 high‐grade glioma patients and showed equal or lower maximum and mean doses to organs‐at‐risk (OARs) compared to VMAT arcs chosen by an experienced dosimetrist.^8^ A separate prospective phase II trial performed automated planning using HyperArc™ (Varian Medical Systems, Palo Alto, CA) noncoplanar VMAT arcs for recurrent head and neck cancers with a cohort of 15 patients.^9^ This research concluded statistically significant increases in dose conformity with increases to maximum doses to OARs that were well below desired planning constraints. Moreover, this work examined moving from two coplanar VMAT arcs to four noncoplanar arcs at couch angles defined by Clark et al.^10^ finding an increase in delivery time of approximately 2 min to be not clinically significant.^9^

Clinical adoption of trajectory optimization has primarily used Varian's HyperArc™ (Varian Medical Systems, Palo Alto, CA), where a specific geometry defined by a clinical class solution of VMAT arcs^10^ is given to begin the treatment planning process for cranial indications. Evaluating this cranial class solution (though not using HyperArc commercial product) compared to patient‐specific noncoplanar VMAT arc optimizations has shown favorable OAR sparing for the latter.^11^, ^12^, ^13^, ^14^

According to a recent review of noncoplanar radiotherapy optimization, there have been few efforts to perform comparisons between coplanar VMAT arcs with noncoplanar VMAT arcs in sites outside of the cranium.^1^ This is supported by earlier work finding noncoplanar VMAT arcs chosen by a human were more efficient to deliver than static noncoplanar IMRT ports for liver stereotactic body radiation therapy (SBRT).^15^ However, it is impractical to ensure a human operator consistently and optimally selects noncoplanar arcs due to the vast degrees of freedom that must be considered.^15^

Previous noncoplanar optimizations have cataloged the geometric suitability of delivering specific BEVs based on the amount of overlap with the PTV in a volumetric projection at isocentre.^3^, ^4^, ^11^, ^12^, ^14^, ^15^ To build in dosimetric tolerance of individual OARs, maximum dose weighting factors have been applied based on published maximum dose tolerance values.^12^, ^14^ This is suitable for the use case of OARs inside the cranium due to their serial functionality and maximum dose constraints. For example, every BEV that contains brainstem overlap can be equally weighted by its maximum dose constraint as the biological response should be the same if any functional subunit of the brainstem receives a dose larger than its maximum tolerance. However, in extracranial radiotherapy such as liver SBRT, considerations must be made for the serial nature of abdominal OARs such as the bowel, stomach, and duodenum, while also considering the parallel nature of the liver and kidneys.

In this research, we propose a methodology for automatically optimizing the selection of noncoplanar arcs for liver SBRT that considers serial and parallel extracranial OARs and preliminary considerations for delivery efficiency. The method considers OARs in three categories and uses geometric raytracing to calculate distinct costs using overlap with the PTV in the radiation beams‐eye‐view (BEV) for each category. This is then combined with the trajectory sampling metric, mean arc distance (MAD), to generate noncoplanar arc trajectories. Considering these geometry metrics with the delivery efficiency metrics of the number of arcs and arc length allows a stochastic algorithm to choose optimized arc trajectories bound by patient‐specific collision zones.

Although generalizable to any anatomical site in the body and extendable to dynamic axes optimization, the aim of this research study was to propose a methodology that would introduce minimal changes to current clinical workflows. In this way, future clinical trials with noncoplanar methodologies can be facilitated.

## 2 METHODS

### 2.1 Raytracing through BEV projection

To calculate a cost associated with the overlap in the BEV between PTV and OARs, the ray‐triangle intersection method of Möller and Trumbore^16^ offers an efficient methodology to trace through many large OARs for each unique aperture. This methodology requires data in the form of meshes/triangulations. Briefly, the method begins with a ray, parameterized according to its source origin and direction. Triangles are defined using edges and vertices, thus structures in the form of triangular meshes are required for the method. Instead of measuring intersections with individual points, ray‐triangle intersections using vector algebra require fewer calculations.^16^ Clinical structures (body contour, PTV, OARs) were exported from the treatment planning system as 3D point clouds. The body contour was preprocessed to remove discontinuities and triangulated in MATLAB (R2022a, The MathWorks Inc., Natick MA).

Overlap was tested for every unique BEV by projecting the PTV and OAR contours to a plane at isocentre. If there was no overlap, the BEV was assigned a cost score of 0; however, if there was overlap, the assigned cost calculation was dependent on whether the OAR was serial or parallel.

### 2.2 Encompassing OARs

Encompassing OARs were defined for the purposes of this work as the OARs which contain the PTV, in this case, the liver. Previous literature using BEV cost calculations has not explicitly considered these OARs as there is overlap with every BEV.^11^, ^12^, ^13^, ^14^ To overcome this, we assign a depth cost specifically to the liver minus GTV OAR. Rays were traced from the source to a gridded 2D projection of the PTV at isocentre, with a fixed resolution of 1 ray per 5 mm. Depth was calculated by measuring intersection distances along ray lines traced from the source through each point on the projection grid. The average PTV entrance depth was subtracted from the average OAR entrance depth, yielding a BEV‐specific cost of delivering radiation through depths in the liver. This is summarized by Equation (1).

(1) $$L\left(C,G\right)\;=\left(\frac{1}{N}\sum_{i=1}^{N}{\left(p-l\right)}_{i}\right)\;$$

where *L (C, G)* is the cost of delivering radiation to the liver at the BEV unique to couch angle *C* and gantry angle *G*. This equation contains *l*, the entrance depth to the liver along a ray *i*, and *p_,_* the entrance depth to the PTV at the depth along the same ray. We expect *p *> *l* as the PTV is in the liver, where smaller values indicate the PTV in close proximity to the liver, and larger values indicate regions where more liver must be traversed to reach the PTV. Here, *N* denotes averaging the differences between entrance depths and corresponds to the total number of rays. This yields a 2D cost map with each pixel corresponding to a depth‐informed cost of delivering to the PTV through the liver.

A calculation was then performed to broaden the solution space, while ensuring geometric regions of large depths (thus high cost) were considered for avoidance. The mean value of this 2D cost map was chosen as a threshold. This ensured cost was attributed to paths with long lengths through the liver that exceeded the mean depth calculated for all apertures. Finally, the map was normalized to its maximum value to facilitate combining with cost from all other OARs.

### 2.3 Parallel OARs

The parallel OARs used in these optimizations were the heart, left kidney, and right kidney. These parallel structures did not contain the PTV and do not have maximum dose constraints according to RTOG1112.^17^ To calculate the BEV cost when these OARs overlap with the PTV, the same raytracing methodology was applied to a different calculation than the encompassing liver OAR. The calculation followed the methodology of Lincoln et al.^18^

This is summarized for a single ray by Equation (2).

$$P\;\left(C,G\right)=\frac{AUC_{p}}{AUC_{T}}\;$$

$$\mathrm{AU}C_{p}=\;\underset{d_{1}}{\int^{d_{2}}}\mathrm{PDD}\left(x\right)\mathrm{dx}$$

(2) $$\mathrm{AU}C_{T}=\underset{0}{\int^{30}}\mathrm{PDD}\left(x\right)\mathrm{dx}\;,$$

where the BEV cost for a parallel OAR *P*, at the BEV unique to couch angle *C* and gantry angle *G* depends on the ratio of the partial area under the curve (*AUC_p_*) defined by OAR entrance distance *d_1_* and exit distance *d_2_* to the total area under the PDD curve (*AUC_T_*). The PDD was measured from depth 0 to 30 cm, which was used to interpolate body entrance and exit points and give a constant value for *AUC_T_*_._ The cost is summed over all rays and then normalized for each parallel OAR.

### 2.4 Serial OARs

The serial OARs used in these optimizations were the stomach, duodenum, and spinal cord. They each have maximum dose constraints according to RTOG1112.^17^ Before exporting from the TPS, each OAR was expanded with a 5‐mm margin to create a planning risk volume (PRV), which was used in the BEV optimization. As serial OARs differ biologically from parallel and parallel‐encompassing OARs, a separate BEV cost was calculated using the same raytracing algorithm.

The degree to which serial OAR BEV overlap with the PTV necessitates dose‐sparing effort depends on the spatial proximity between the two structures. Ensuring rapid dose fall‐off outside the PTV is a characteristic of SBRT treatment planning, therefore cost was only calculated for serial OARs if they fell within a fall‐off region specified by considering the OARs specific dose constraint (*D_OAR_*). This is summarized in Equation (3).

(3) $$A_{fall}\left[\mathrm{mm}\right]\;=\frac{100\left[\%\right]*\left(1-\frac{D_{OAR}\left[\mathrm{Gy}\right]}{D_{R_{x}}\left[\mathrm{Gy}\right]}\right)}{G\;\left[\frac{\%}{\mathrm{mm}}\right]}\;$$

where *A_fall_* is the necessary fall‐off distance for inclusion in the cost function at an achievable gradient *G*. In this work, *G* was set equal to 5% per mm, a more conservative estimate of an achievable gradient than previous work.^11^ This value is arbitrary but based on stereotactic treatment planning goals of achieving a rapid fall‐off outside the target. The regions of serial OARs that fall within this gradient, should be considered due to their maximum dose (0.03 cc) tolerances. Only points on a serial OAR less than or equal to *A_fall_* are included in the BEV cost calculation. The closest distances between the PTV and each serial OAR were measured before raytracing. All points less than or equal to *A_fall_* were triangulated to create an avoidance structure corresponding to the serial OAR. Any point in an OAR greater than *A_fall_* was not considered in the calculation of cost. The cost of avoiding this structure followed Equation (2) and was repeated for each serial OAR. In cases where the serial partial avoidance structure could not be triangulated into a continuous convex volume, the cost was assigned a value of zero. The underlying assumption here was that if there were insufficient points to produce a triangular mesh volume, then the contribution to overall cost was insufficient as well. This problem only occurred for a handful of BEV when there were approximately <10 points. In general, whenever there are concavities that arise due to structure curvature, the closest entrance distance from the source is taken as *d_1_* and the farthest exit distance from the source is taken as *d*_2_.

### 2.5 Constructing the total BEV cost map

Combining all OARs to create the total BEV cost map followed Equation (4).

(4) $$E\;\left(C,G\right)=\;L\left(C,G\right)+P\left(C,G\right)+S\left(C,G\right)$$

where the total cost is the sum of the normalized encompassing liver depth cost *L* (Equation 1), the parallel OARs overlap cost *P* (Equation 2), and the serial OAR avoidance cost *S* (application of Equation 2 to serial OARs). This total BEV cost map underwent a final normalization, and then patient‐specific collision zones were added in a modification to previously published techniques.^19^

**FIGURE 1:**
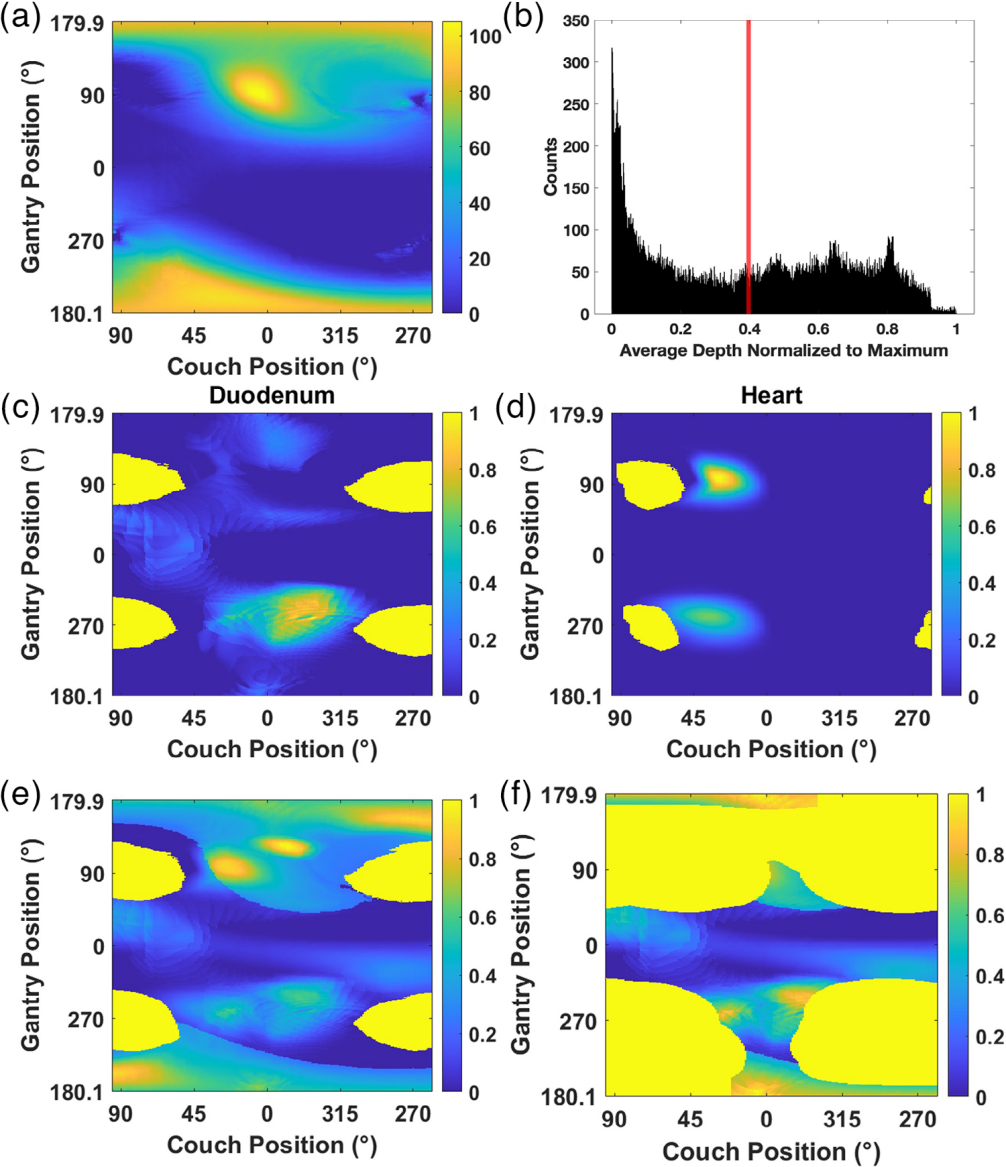
(a) The raw depth cost values extracted for the liver minus GTV OAR with colorbar scale measured in millimeters [mm]. (b) Histogram of depth in (a) normalized to the maximum, with a red line denoting the mean depth value. All values less than the mean depth of (a) (to the left of red line on (b)) are then set to zero. (c) Shows an example serial OAR BEV cost map for the duodenum of the same case, and (d) shows an example parallel OAR BEV cost map for the heart of the same case. (e) Shows the resulting combination of all OARs without collision zones. The brightest yellow regions of (c–e) indicate raytracing occurring through superior/inferior limits of the CT set. (f) The final BEV cost map showing the combination of all OARs with collision zones.

An example case undergoing all steps of BEV cost map construction is shown in Figure 1.

The dark blue regions on the map of Figure 1a represent areas of shallow PTV depth inside the liver while lighter yellow demonstrates deeper PTV depths as calculated by Equation (1). In Figure 1c–f, dark blue regions correspond to lower normalized BEV cost scores as calculated by Equations (2)–(4), while the lighter yellow regions are higher normalized BEV cost scores. The brightest yellow regions of Figure 1c–e represent where rays are traced through the most superior or inferior slices of the CT set. In most cases, these would correspond to tracing through the head/arms (superior) or the legs (inferior), which we want to avoid at all costs and hence assign a cost of infinity. According to Figure 1f, raytracing that occurs through superior/inferior limits of the CT scan is covered by the patient‐specific collision zone, which is also assigned infinite cost.

### 2.6 Patient‐specific arc trajectories

The BEV cost maps that quantify priority avoidance of OARs were used to create patient‐specific arc trajectories by first creating a candidate pool of deliverable arcs, and then performing a stochastic search to find optimized arcs.^18^ It was deemed important that the candidate pool contain a mix of long and short arcs. This gives the arc selection algorithm flexibility in balancing lower spatial sampling if short arcs with low cost are used with longer arcs that will increase spatial sampling at the expense of potentially higher BEV cost.^18^

A metric developed by MacDonald et al. called MAD allows for quantitative comparison of 4π spatial sampling between any trajectory.^20^ Using a sphere with equally spaced points, control points of a trajectory are mapped onto the same sphere. Arc distance allows for these positions to be related using Equation (5):

(5) $$\alpha_{j,k}=\tan^{-1}\left(\frac{\vec{a_{j}}\times \vec{b_{k}}}{\vec{a_{j}}\cdot \vec{b_{k}}}\right)\;$$

where $\alpha_{j,k}$ is the angle between a unit vector from the origin to a control point *j* along the given trajectory (*a*) and the unit vector from the origin to a sampling point *k* on the sphere (*b*). This angle is directly proportional to arc length according to the unit sphere's radius. The closest control point to a given sampling point minimizes $\alpha_{j,k}$ and MAD is calculated according to Equation (6):

(6) $$MAD\;=\;\mu_{\alpha }=\frac{1}{n}\;\sum_{i\;=\;1}^{n}\alpha_{i,\min}$$

where *n* is the total number of sample points. Therefore, lower MAD implies greater 4π spatial sampling.

Approximately 5000 candidate arcs were created for each BEV cost map by the method of Lincoln et al.^18^ The stochastic search begins by choosing a random sampling of subarcs from the candidate pool to generate a trajectory. The MAD for this specific trajectory was calculated and stored with the trajectory‐specific BEV cost. The stochastic search was subjected to 100 000 permutations, yielding a spectrum of MAD and BEV cost values for simultaneous optimization.

An optimized trajectory of high quality should have sufficient geometric sampling (low MAD), minimal overlap with OARs in the BEV (low BEV cost) and consider delivery efficiency (low number of arcs and reasonable number of control points). A tradeoff exists between these parameters where the lowest MAD will have many short arcs to sample the space, while the lowest BEV score is the shortest arc in the region of lowest overlap, having poor sampling. To optimize MAD, BEV cost, and arc number from this filtered set, the following algorithm was applied:

1. The minimum number of arcs was chosen to be 2, while the maximum number of arcs was chosen to be 10.
2. For each potential number of arcs (2–10), find solutions that have a MAD score within the bottom 5% (empirically chosen) of that subset.
3. Sort the entire subset found in (1) by the quadrature sum of BEV cost and MAD.
4. The optimized arc trajectory minimizes the value found in (3).

**FIGURE 2:**
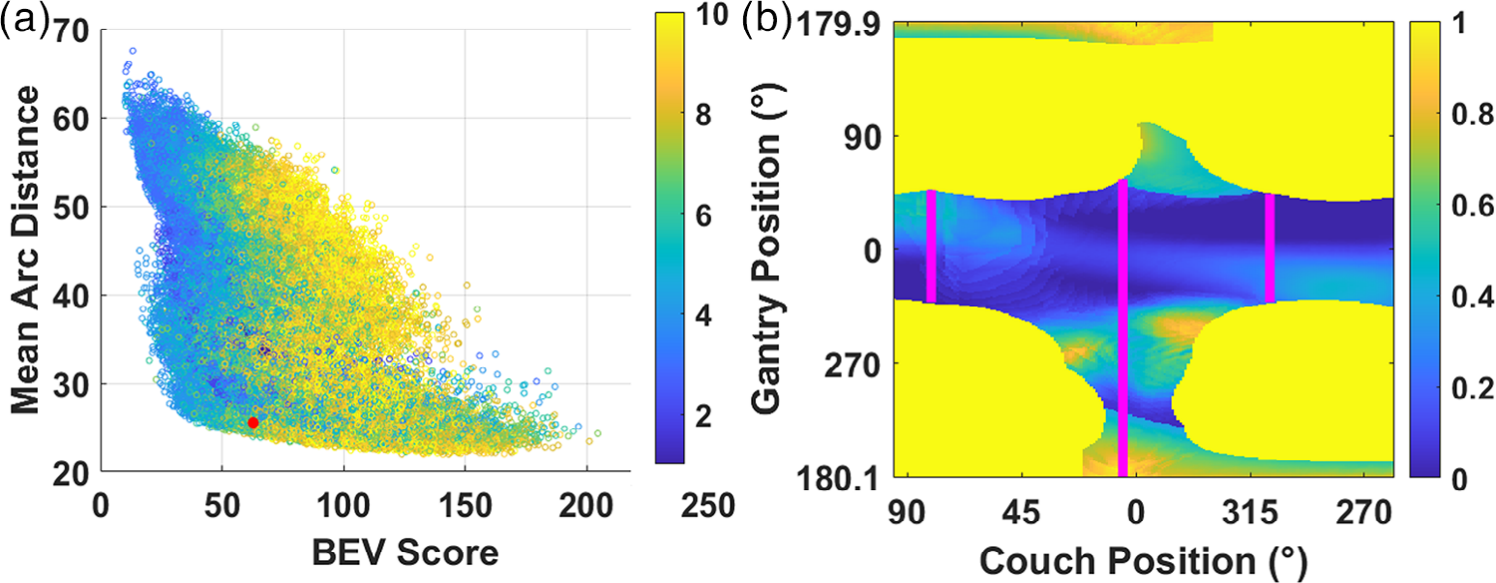
(a) BEV score plotted against MAD for 100 000 random arc trajectories. Dots denoted by the colorbar indicate the number of arcs in the solution ranging from 2 to 10. The red dot shows the scores for the optimized arc trajectory that was chosen. (b) The example BEV cost map with collision zones from Figure 1f with the optimized arc trajectory from 2a shown in magenta lines.

Therefore, each contribution to a high‐quality trajectory arc set was considered in the optimization. This algorithm will inherently prioritize solutions with a lower number of arcs that are correlated with a lower BEV cost, and thus a lower quadrature sum. Choosing the bottom 5% of MAD for each number of arcs, instead of all trajectories, ensures that solutions are biased away from a high number of arcs. Figure 2 shows an example of the arc trajectory chosen based on the spectrum of solutions for the example case shown in Figure 1.

The red point representing the solution is positioned away from the bottom left‐hand corner such that it satisfies a lower MAD, while trying to maintain a low BEV score and low number of arcs. It is readily seen from Figure 2 that the patient‐specific collision zone prohibits a full (uninterrupted gantry span top to bottom) coplanar or noncoplanar arc. Full coplanar/noncoplanar arcs were not used in this study as they were always limited by collision zones. These patient‐specific collision zones are based on an isocentre placed in the center of mass of the target (our institutional practice), if an isocentre is placed in the middle of the patient the gantry will clear a full rotation. Placing an isocentre in the middle of the patient to avoid collisions could make achieving the target dose more difficult due to larger contributions off‐axis and more monitor units may be required. Details of the starting angle, ending angle and number of arcs for all noncoplanar plans are found in Table 2 of the results section.

### 2.7 Treatment planning

Treatment planning was performed retrospectively on 16 patients selected randomly who previously received liver SBRT at our institution. Patient cases were anonymized, and their use was approved by the Nova Scotia Health Research Ethics Board. Of these 16 patients, eight were designated small targets (PTV < 50 cc) while eight were designated large targets (100 cc < PTV < 500 cc). Table 1 summarizes the treatment plan characteristics, with target positions characterized as being central or noncentral.

**TABLE 1:** Target volume descriptions for the 16 patients used in this study including prescription dose, target position, and fractionation.

Patient ID	Target volume (cc)	Target position in liver	Prescription (Gy)/fractions
1	47.8	Noncentral	54/5
2	35.7	Noncentral	35/5
3	38.7	Noncentral	45/5
4	20.6	Central	45/5
5	5.7	Noncentral	50/5
6	43.3	Noncentral	50/5
7	47.9	Central	45/5
8	13.9	Noncentral	50/5
9	432.8	Central	30/5
10	115.1	Noncentral	54/5
11	171.5	Central	40/5
12	278.8	Central	45/5
13	145.1	Noncentral	50/5
14	167.1	Central	30/5
15	263.5	Central	27.5/5
16	217.6	Central	40/5

As the OARs included in VMAT optimization for liver SBRT treatment planning are at the discretion of the prescribing radiation oncologist based on proximity to the PTV, the number can vary between patients. Introducing noncoplanar arcs has the potential to include OARs that were not originally considered. Therefore, a standardized set of OARs was contoured by two expert dosimetrists with extensive experience contouring OARs for liver SBRT. These OARs were contoured for each patient corresponding to the OARs used in the BEV cost map construction described in Sections 2.2–2.4.

Each patient was replanned with two VMAT plans differing only by arc geometry used. The first used a clinical arc template of two coplanar arcs at couch position 0° spanning 200° from gantry position 20° to 180° in both clockwise and counterclockwise directions, with complementary collimator angles (15° and 345°, respectively). The second plan used the optimized arcs described in Section 2.6.

VMAT optimization was performed for all plans by two medical physicists with considerable experience planning clinical liver SBRT. The Eclipse treatment planning system (Varian Medical Systems, Palo Alto CA) version 15.6 was used with photon optimizer (PO) version 15.6 and the Acuros External Beam (AXB) algorithm for dose calculation.^21^ As per institutional standards, the prescription dose for each plan was prescribed to the 90% isodose. Standardized VMAT optimization parameters were automatic normal tissue optimization (NTO = 175) and an AXB dose calculation grid resolution of 1.5 mm. Specific priorities on the PTV, OARs, and/or tuning structures were then modified as needed to aid with target coverage, OAR sparing, and low dose spread. These priorities were adjusted depending on local anatomy with the effect of meeting the objectives outlined in RTOG1112.^17^ For example, if RTOG1112 objectives were not met for the clinical solution plan, they were addressed by adjusting structure weights in the optimization or creating tuning structures to control region‐specific dose gradients.

### 2.8 Plan comparison

The plan metrics found in RTOG1112^17^ were used as the basis for comparison between arc geometries. These guidelines are used in clinical institutional practice for treating liver SBRT. Maximum dose (to 0.03 cc) metrics and dose–volume objectives specific to RTOG1112^17^ were evaluated for the OARs considered in the treatment planning process. PRV stomach, duodenum, heart, and spinal cord were considered with maximum dose tolerances <30 Gy. The dose–volume tolerances for the liver included the volume receiving 10, 18, and 21 Gy as well as the effective liver volume. Target metrics based on the Paddick conformity and gradient index (CI, GI)^22^, ^23^ were evaluated according to Equations (7) and (8), respectively. Target coverage was also evaluated in terms of the maximum dose of 0.03 cc and mean dose to the target.

(7) $$CI\;=\frac{V_{T,ref}}{V_{T}}\;\times \frac{V_{T,ref}}{V_{ref}}$$

where *V_T,ref_* is the volume of the target (*T*) that receives a dose greater than or equal to a reference dose. *V_T_* is the volume of the target, and *V_ref_* is the reference isodose volume. In this work, *V_ref_* is equal to the prescription isodose volume given in Table 1.

(8) $$GI\;=\frac{V_{50\%R_{x}\;}}{V_{R_{x}}}\;$$

where the *GI* is defined as the ratio of the volume receiving half of the prescription isodose to the volume receiving the prescription isodose.

An in‐house developed ESAPI script extracted the relevant DVH metrics inside the TPS to a tabular format. The table was then exported into MATLAB to perform final analysis and statistical testing. The Wilcoxon Signed‐Rank test (two‐tailed) is a nonparametric statistical test that was used as the data were not normally distributed. Comparisons between plan quality metrics of the two arc selection methods were made with a significance level set at *p* < 0.05.^24^

## 3 RESULTS

Details of the noncoplanar arc geometries are shown in Table 2. Most solutions included a combination of long (>100°) and short (<100°) arcs. Short arcs were included at a greater frequency for solutions with a greater number of arcs in total. The longest arc of 277° was found for test patient 12 in a solution that included three arcs in total.

**TABLE 2:** Details of noncoplanar arc geometries for all plans including number of arcs with corresponding arc length, static couch angle, gantry start angle, and gantry stop angle. All angles correspond to the IEC 61217 standard.

Patient ID	Number of arcs (arc length°)	Couch angle	Gantry start	Gantry stop
1	1/7 (39)	72	29	350
	2/7 (35)	350	81	46
	3/7 (51)	295	25	334
	4/7 (127)	22	307	180
	5/7 (34)	313	214	180
	6/7 (36)	338	179	143
	7/7 (31)	33	179	148
2	1/4 (86)	87	45	319
	2/4 (184)	346	95	271
	3/4 (151)	17	49	258
	4/4 (42)	348	222	180
3	1/4 (34)	346	179	145
	2/4 (174)	22	354	180
	3/4 (58)	272	28	330
	4/4 (42)	309	212	180
4	1/3 (174)	343	78	264
	2/3 (73)	265	35	322
	3/3 (35)	358	179	134
5	1/4 (85)	82	44	319
	2/4 (31)	343	211	180
	3/4 (143)	21	44	261
	4/4 (174)	341	82	295
6	1/4 (192)	346	91	259
	2/4 (81)	94	42	321
	3/4 (111)	31	41	290
	4/4 (34)	344	214	180
7	1/7 (73)	73	37	324
	2/7 (31)	349	174	143
	3/7 (72)	59	33	321
	4/7 (32)	13	179	147
	5/7 (64)	283	30	326
	6/7 (80)	347	294	214
	7/7 (124)	27	36	272
8	1/5 (79)	21	259	180
	2/5 (100)	36	34	294
	3/5 (58)	94	29	331
	4/5 (31)	338	179	148
	5/5 (37)	316	217	180
9	1/4 (36)	9	179	133
	2/4 (139)	345	90	311
	3/4 (41)	21	221	180
	4/4 (72)	264	32	320
10	1/4 (40)	322	220	180
	2/4 (217)	344	37	180
	3/4 (68)	73	33	325
	4/4 (123)	33	31	268
11	1/5 (83)	86	44	321
	2/5 (42)	349	222	180
	3/5 (86)	47	36	310
	4/5 (81)	307	42	321
	5/5 (190)	358	117	287
12	1/3 (277)	351	97	180
	2/3 (86)	309	44	318
	3/3 (85)	269	40	315
13	1/4 (218)	355	38	180
	2/4 (97)	355	179	82
	3/4 (31)	318	211	180
	4/4 (66)	80	34	328
14	1/3 (42)	20	222	180
	2/3 (79)	82	41	322
	3/3 (179)	346	85	266
15	1/6 (37)	22	247	210
	2/6 (63)	282	30	327
	3/6 (101)	339	36	295
	4/6 (37)	353	178	141
	5/6 (48)	326	218	180
	6/6 (77)	45	34	317
16	1/3 (235)	7	55	180
	2/3 (85)	311	43	318
	3/3 (88)	80	46	318

**FIGURE 3:**
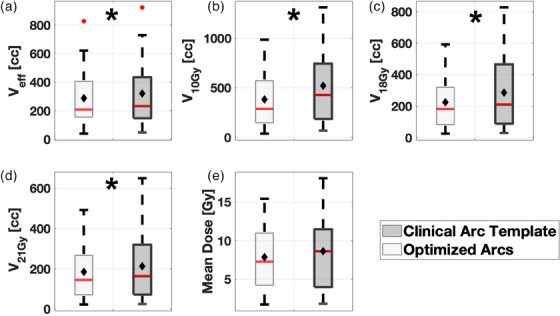
(a) Liver metric results for effective liver volume (*V_eff_*). Percentage of the liver volume receiving 10 Gy is shown in (b). The dose–volume results are shown for 18 Gy (c), 21 Gy (d), and mean dose (e). Light gray boxes denote the optimized arc solution, while dark gray boxes represent the clinical arc template. The median is given as the red line inside each box, while the mean is denoted as filled black diamonds. Outliers are illustrated with filled red circles. Statistically significant differences (*p* ≤ 0.05) are denoted as black stars.

Figure 3 shows a boxplot of the liver metrics for both methods of arc selection. The OAR used for measurements of these metrics was “Liver—GTV” (liver minus GTV).

From Figure 3a to Figure 3e, all liver metrics considered in this study were reduced using optimized arcs, with one reduction not meeting the criteria for statistical significance: the mean dose to the liver reduction of 1.34 (2.55) Gy (*p* = 0.06). Figure 3a demonstrates that using optimized arcs results in a statistically significant reduction in the effective liver volume of 24.5 (88.5) cc on (*p* = 0.04). Figure 3b shows a statistically significant reduction in V10 Gy of 140.6 (295.8) cc (*p* = 0.001). From Figure 3c to Figure 3d, on average, the optimized arcs decreased the volume receiving 18 and 21 Gy by 28.2 (230.6) cc (*p* = 0.002) and 18.5 (155.5) cc (*p* = 0.04), respectively, compared to the clinical arc template.

**FIGURE 4:**
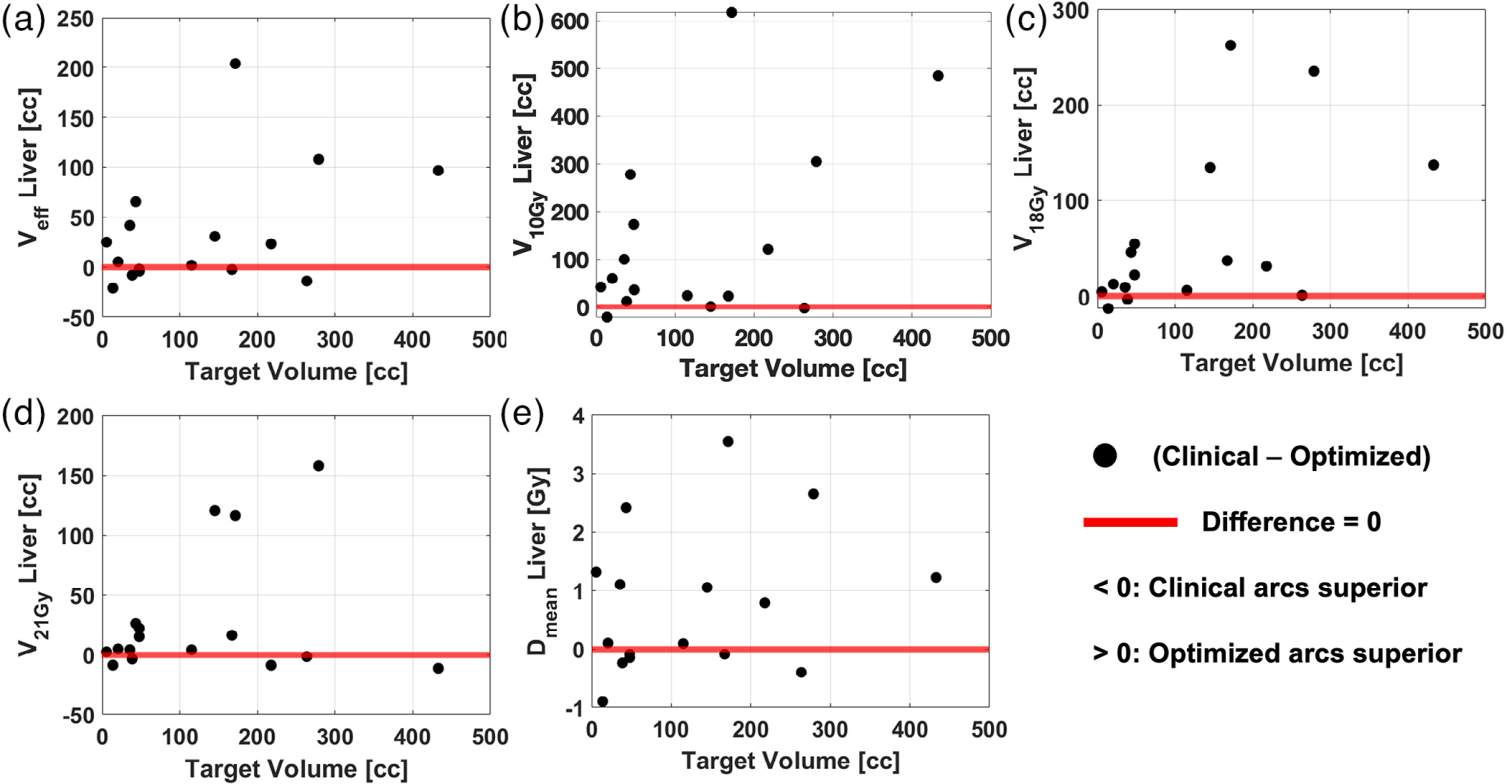
Difference between clinical and optimized arcs for each metric corresponding to Figure 3a–e was plotted as a function of target volume. Black dots indicate the difference in subtracting the metric using clinical arcs from the same metric using the optimized arc solution. Red lines indicate the baseline difference equal to zero where there would be no quantifiable difference in metric based on the method for geometric arc selection.

Figure 4 expands on the differences in the liver metrics between geometric arc selection techniques by displaying the results for all cases of the quantitative differences between arc selection methods as a function of target volume. All points below the red line indicate where the clinical arcs offered superior liver sparing, while the points above the red line indicate where optimized arcs offered superior liver sparing.

Greater numbers of points above the red line for all metrics demonstrated by Figure 4a–e give insight to the significant reductions in liver metrics shown in Figure 3a–e. The largest comparative reduction in liver volume using optimized arcs is shown in Figure 4c where V18 Gy was reduced by 262.2 cc in one case for a target volume of 171.5 cc. Conversely, the largest comparative increase in liver volume using optimized arcs is shown in Figure 4a where *V_eff_* was increased by 21.2 cc for a target volume of 13.9 cc. Pearson correlation coefficients were computed for each metric yielding weak correlations each below a significance threshold of 0.7. The strongest correlation was found for V18 Gy at *R* = 0.54.

**FIGURE 5:**
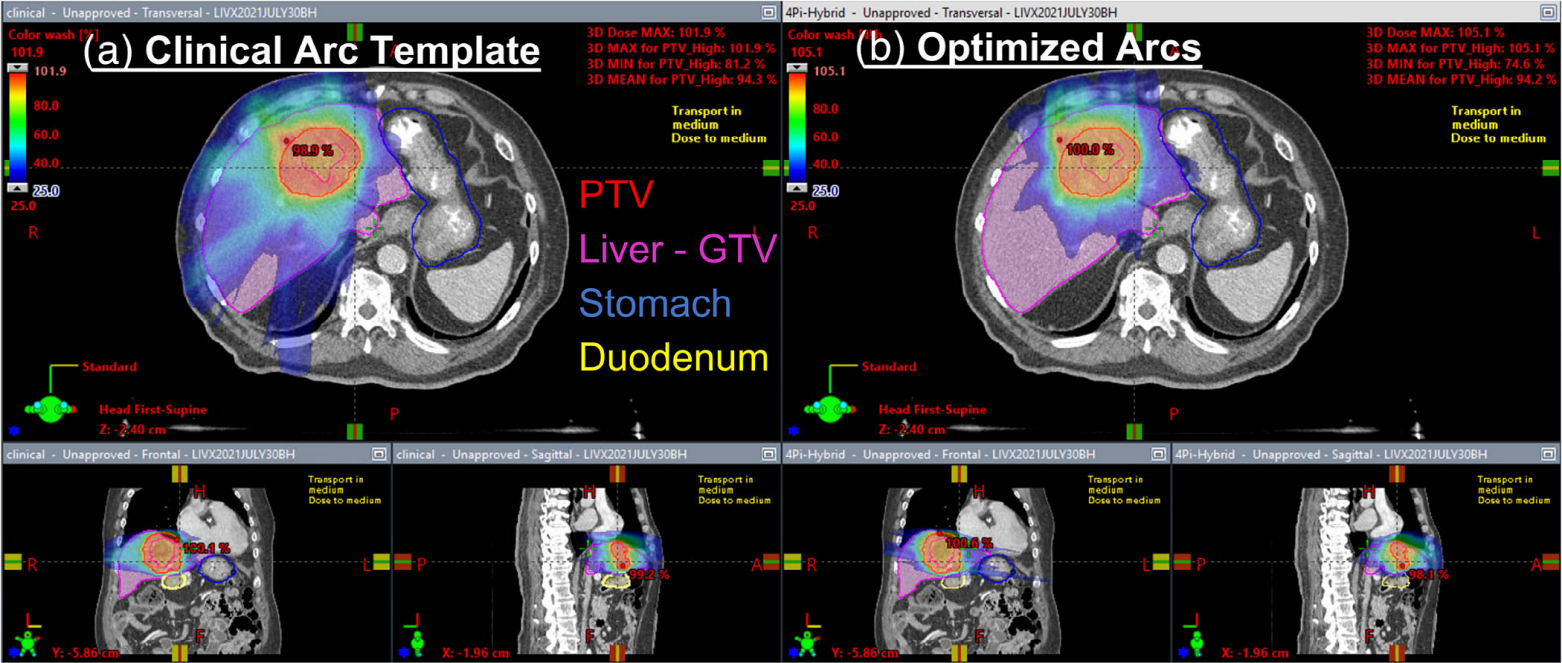
Dose distribution for an example case. The plan using the clinical arc template (a) is shown on the right‐hand side and the plan using the optimized arcs is shown on the left‐hand side for the same slices. Contoured structures corresponding to their contour colors in the TPS are shown. The PTV was contoured in red, the liver minus GTV was contoured in magenta, the stomach was contoured in blue, and the duodenum was contoured in yellow. The lower limit of each dose wash was set to 25%.

Figure 5 displays the 3D dose distribution for an example case (test patient 12, *R_x_* = 45 Gy/5 fx). Figure 5a shows the 25% isodose splaying throughout the majority of the Liver—GTV volume. Figure 5b shows the 25% isodose concentrated into a smaller volume inside the liver.

**FIGURE 6:**
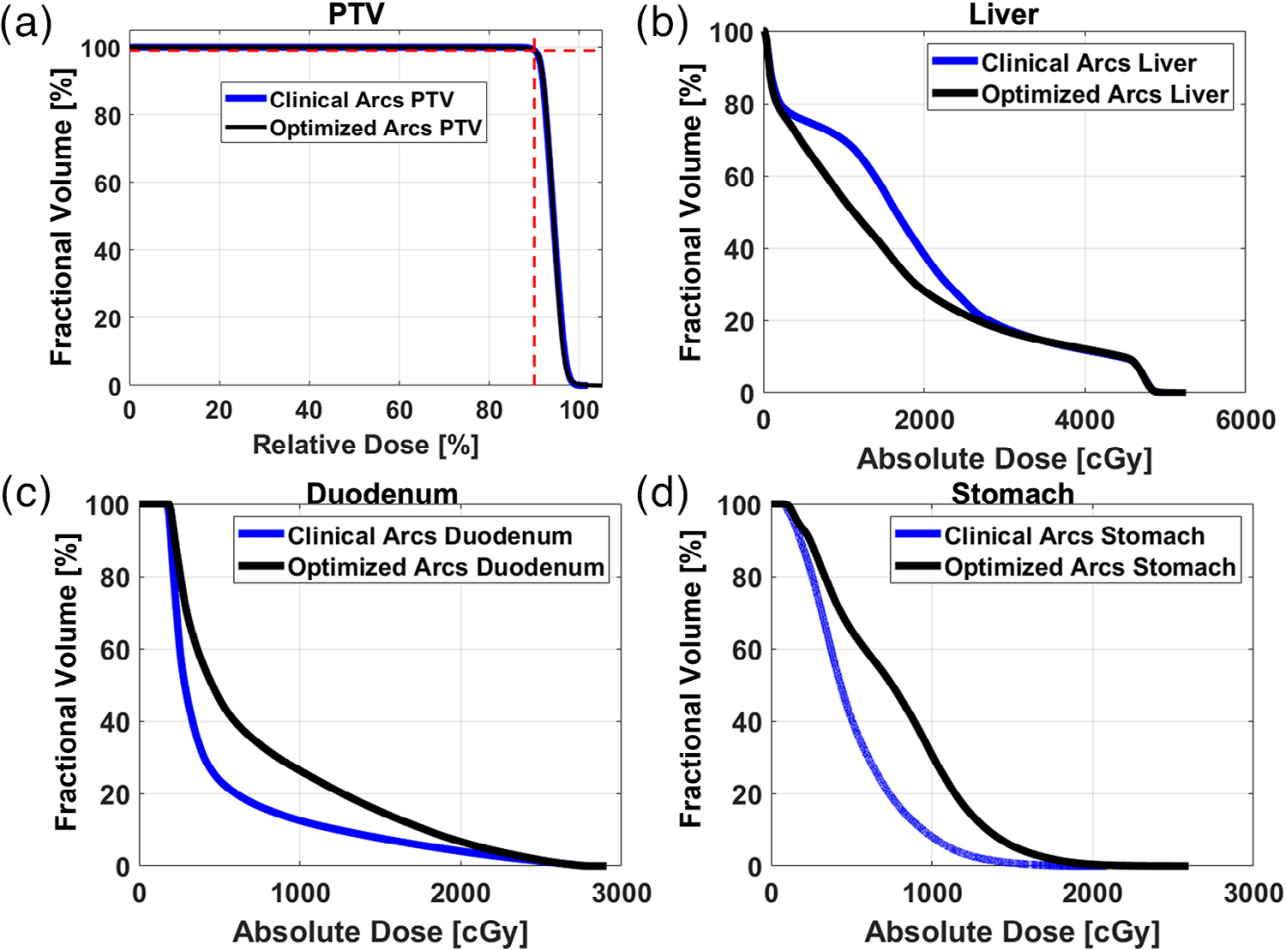
Example DVH for PTV (a), liver minus GTV (b), PRV duodenum (c), and PRV stomach (d). Blue lines denote the DVH for the clinical arc template, while black lines denote the DVH for the optimized arcs solution. The red dashed line of Figure 6a shows the normalization point of the prescription dose at 99% volume covered by the 90% isodose (100% of the *R_x_*).

Figure 6 shows an example dose volume histogram (DVH) for the case of Figure 5 with the same OARs.

From Figure 6a, comparable PTV coverage was found for this example case where blue and black DVH curves are approximately equal. The DVH of Figure 6b shows similarities between methods of arc selection except at doses approximately below 2500 cGy where the optimized arcs demonstrate lower volume irradiated. Conversely, larger irradiated volumes using the optimized arcs were found for both the stomach and duodenum, however each remained below their respective dose constraints.

**FIGURE 7:**
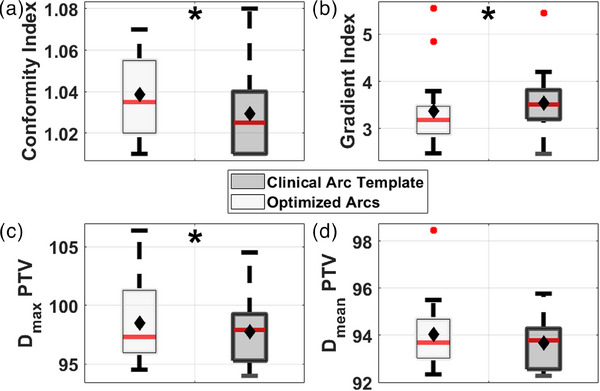
Conformity index of the target volume (a), gradient index (b), maximum dose inside the target (c), and mean dose inside the target (d). Light gray boxes denote the optimized arc solution, while dark gray boxes represent the clinical arc template. The median is given as the red line inside each box, while the average is denoted as filled black diamonds. Outliers are illustrated with filled red circles. Statistically significant (*p* ≤ 0.05) differences are denoted as black stars.

Target metrics are shown in Figure 7. Three metrics from Figure 7 were statistically significant. Figure 7a shows the conformity index that increased by 0.01 (0.01) using optimized arcs (*p* = 0.006). Figure 7c shows the maximum dose inside the PTV also increased by 0.6 (1.3) % (*p* = 0.04) when using optimized arcs. These differences were well below the tolerance of 1.2 for CI and 108% for PTV *D_max_*, respectively. From Figure 7d, optimized arcs increased the mean PTV dose by 0.1 (2.6) %, which was not deemed statistically significant. Figure 7b shows that the gradient index was statistically significantly reduced using optimized arcs by 0.3 (0.1) (*p* = 0.03).

**FIGURE 8:**
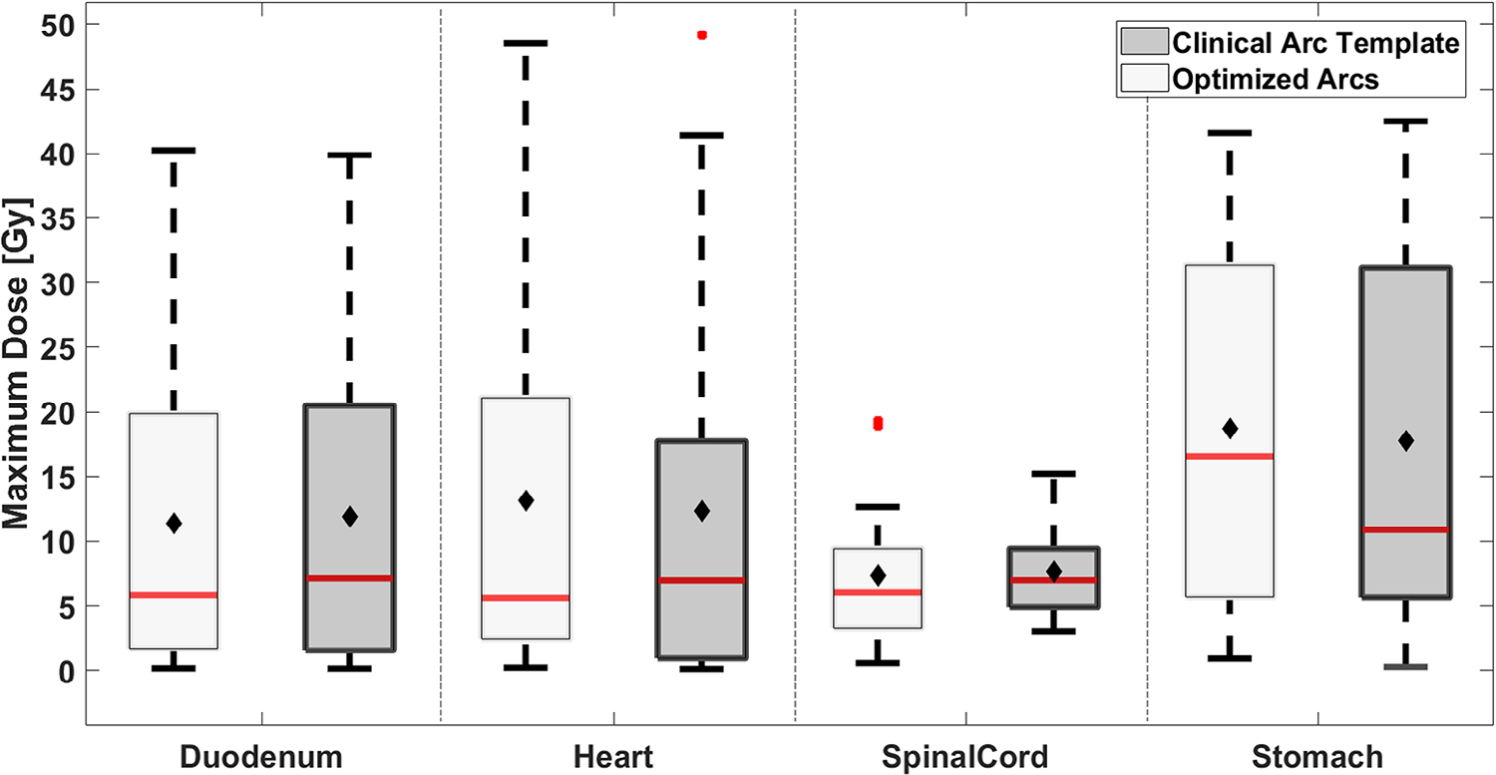
Maximum dose to 0.03 cc for OARs considered in the optimization with maximum dose constraints. Light gray boxes represent the optimized arc solution, while dark gray boxes represent the clinical arc template. The median is given as the red line inside each box, while the average is denoted as filled black diamonds. Outliers are illustrated with filled red circles. Statistically significant differences are illustrated with black stars.

From Figure 8, a maximum dose increase was found for the PRV stomach of 5.7 (1.6) Gy. Dose reductions were found for the PRV duodenum, heart, and PRV spinal cord by using optimized arcs of 1.3 (0.4) Gy, 1.4 (0.7), and 0.9 (6.6) Gy. None of these increases or reductions was statistically significant.

**FIGURE 9:**
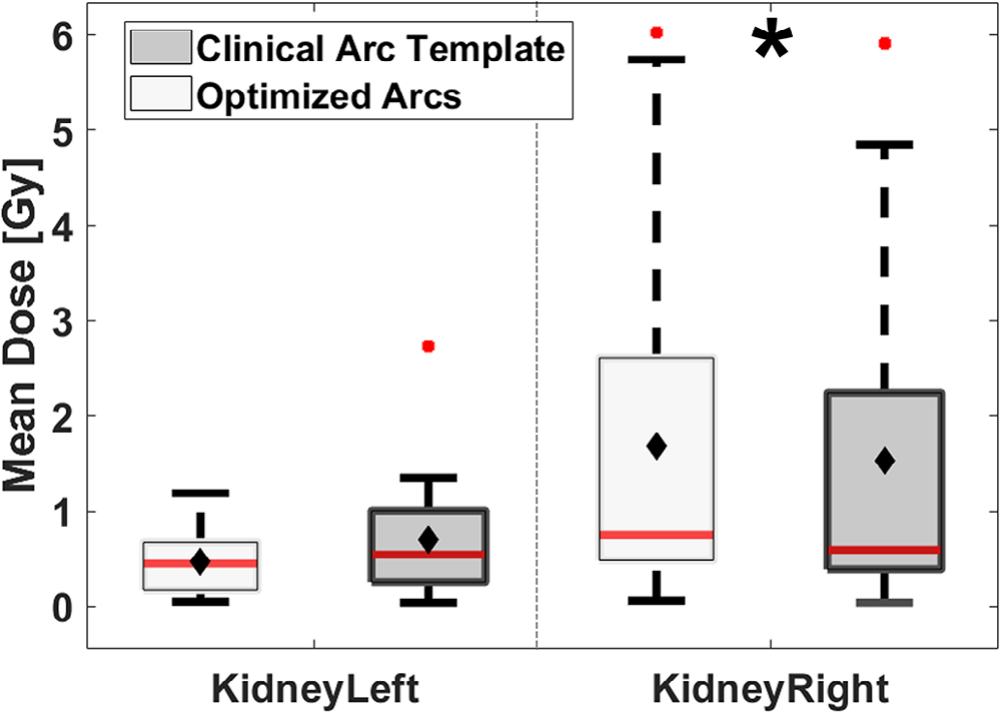
Left and right kidneys were considered in the optimization with mean dose constraints. Light gray boxes represent the optimized arc solution, while dark gray boxes represent the clinical arc template. The median is given as the red line inside each box, while the average is denoted as filled black diamonds. Outliers are illustrated with filled red circles. Statistically significant differences are illustrated with black stars.

From Figure 9, one statistically significant dose increase was found for the right kidney of 0.2 (0.1) Gy (*p* = 0.03) on average from using optimized arcs. The left kidney mean dose was reduced by 0.1 (1.6) Gy, however, this reduction was not statistically significant.

## 4 DISCUSSION

In this work, a methodology was presented that incorporates the nature of an OAR's specific functional subunits into the construction of a BEV cost map. These considerations are more pertinent in extracranial sites, such as the liver, as compared to the cranium, and are especially important in hypofractionated dosing regimes. Combining the unique avoidance priorities of serial, parallel, and parallel‐encompassing OARs into a single cost map then allowed for a stochastic pathfinding algorithm to search for an optimized solution. Considerations were made in an attempt to choose arc trajectories that optimized delivery efficiency, BEV cost, and trajectory sampling simultaneously. The method was compared to a clinical arc template used in our institution for treating liver SBRT.

On average, in the cohort of 16 patients that were planned with a clinical arc geometry and optimized noncoplanar arc geometry, the analysis shows an overall tradeoff between liver sparing and OAR sparing. This is reflected in the dose reduction results for the liver as shown in Figures 3, 4, 5 and  6B, in contrast to the maximum dose increase results of Figure 8 and the example DVHs of Figures 6c and 6d. However, the extent to this trade‐off is biased towards liver sparing with low average maximum dose increases being found for OARs and large spared volumes being found for the liver.

Moreover, considering noncoplanar optimizations in the literature for liver SBRT, results can be compared.^3^, ^15^ In both works, compared to coplanar VMAT, noncoplanar IMRT was performed with 14–22 beams on a cohort of 10 patients and 20 field IMRT on a cohort of 20 patients, respectively. Woods et al. also chose to compare three to four noncoplanar VMAT arcs that were selected without optimization. In our work, the stochastic optimization chose four arcs on average. They separately found superior OAR maximum point dose reductions using IMRT compared to VMAT. We found agreement with their research as their IMRT OAR doses are comparable to but less than what we found using both optimized noncoplanar and nonoptimized coplanar VMAT arc selection. Liver sparing was quantified in terms of the volume receiving >15 Gy, compared to this research that used V10, V18, and V21 Gy in accordance with RTOG1112.^17^ Both authors quote reductions in V15 Gy on the order of 50–80 cc with IMRT, while this work found median (range) reductions in V10, V18, and V21 Gy of 140 (296), 28 (231), and 18 (155) cc, respectively.

Although noncoplanar IMRT has the capability to reduce doses in liver SBRT and facilitate dose escalation, the number of treatment fields is clinically cumbersome to deliver.^3^, ^15^ Moreover, when considering patient motion throughout the course of treatment, efficient techniques are needed. The optimized arc solutions created in this research apply considerations for delivery efficiency. This tradeoff is readily apparent in the increased maximum doses to OARs compared to the noncoplanar IMRT proposed by Dong et al. and Woods et al. However, similar dose reductions to liver were found in our work with efficient patient‐specific optimized arc geometries. Although outside the scope of this research, results from this study could be of use in terms of generating a class solution using noncoplanar arcs similar to Clark et al.^10^ More than half of the solutions presented in Table 2 include a 3 or 4 arc solution where one or two long arcs are used with one or two short arcs. The long arcs give flexibility for modulation and efficiency, while the short arcs aim to improve 4π sampling and lower the amount of overlapping OARs that are irradiated.

Specific clinical cases that could benefit from this methodology include improving the capacity for liver SBRT retreatments in settings where the effective liver volume constraint is violated. This methodology could also be suited for noncentral lesions in the liver, due to considerations made based on the pathlength through the liver to the target as central lesions have similar pathlengths that may not be filtered out. For all large (>50 cc) noncentral targets superior liver sparing was found, while large central targets had lower magnitude or even inferior liver sparing compared to the template arcs. Small targets (≤50 cc) were more varied with superior liver sparing found for both central and noncentral targets. Notably, plan selection did not control for target centrality, thus there were fewer small central (2/8) lesions than large central (5/8) lesions.

Target volume‐related trends were also investigated in Figure 4. The weak Pearson correlation coefficients are not statistically significant; however, larger dose reductions were found for target volumes >50 cc. Large targets imply greater liver volumes that must be irradiated and thus avoidance methods such as those presented in this work could have potential value.

A limitation of this study includes the simple approximation made when considering thresholding the BEV cost maps specific to the encompassing liver OAR. Opening the solution space to depths less than the mean depth value could be suboptimal in a centrally positioned target where all depths are approximately equal. Furthermore, more sophisticated methods could be applied to combine the individual BEV cost maps. Specifically, the addition of weighting factors to each of the encompassing, parallel, and serial OAR BEV cost maps could bias the solution towards specific OAR avoidance if desired. The method of Wang et al. uses a cost associated with partial volumes,^25^ which could also be applied to improve the BEV cost equation used in our research. Finally, the stochastic arc selection algorithm has the potential to be improved without using an empirically chosen lower bound of MAD‐informed arc selection. Further research is required to understand the relationship between MAD and conformity. Given this information, the solution set has the potential to be expanded at the lower bound corresponding to acceptable clinical conformity which is not known at this point until after treatment planning.

The calculation time for the stochastic arc selection algorithm to generate 100 000 trajectories is approximately 1 h using MATLAB code that was not optimized for usage of the 4CPU on a 2017 Dell XPS 13 laptop. MATLAB is not typically used in applications where efficiency is prioritized, instead languages such as C# could be more efficient and faster.

## 5 CONCLUSION

In conclusion, this research demonstrated a methodology of noncoplanar arc optimization for VMAT as applied to liver SBRT. Considerations for the degree of seriality of specific OARs were incorporated into a BEV cost function, and a stochastic search algorithm chose optimized arc trajectories that considered delivery efficiency, BEV cost, and trajectory sampling using MAD. On average maximum doses to OARs remained below tolerances specified by RTOG1112 for liver SBRT. The average number of arcs (4 ± 1) could be delivered more efficiently than 14–22 field IMRT proposed in the literature given the limitations of current clinical applications. These results showed significant reductions of irradiated healthy liver volumes for a collection of small and large targets compared to VMAT using a coplanar arc template.

## AUTHOR CONTRIBUTIONS

*Study conception*: John Lincoln, Lee MacDonald, Alasdair Syme, and Christopher Thomas. *Treatment planning*: John Lincoln, Lee MacDonald, Lucy Ward, Shelly Johnston, and Christopher Thomas. *Data analysis*: John Lincoln. *Software development (algorithmic design and analysis)*: John Lincoln. *Manuscript preparation*: John Lincoln. *Algorithmic design*: Lee MacDonald, Alasdair Syme, and Christopher Thomas. *Critical review of manuscript*: Lee MacDonald, Lucy Ward, Shelly Johnston, Alasdair Syme, and Christopher Thomas. *Contouring*: Lucy Ward and Shelly Johnston.

## CONFLICT OF INTEREST STATEMENT

The authors declare no conflicts of interest.

## ETHICS STATEMENT

Nova Scotia Health REB File No: 1025509.
